# “Hand hygiene perception and self-reported hand hygiene compliance among emergency medical service providers: a Danish survey”

**DOI:** 10.1186/s13049-019-0587-5

**Published:** 2019-02-05

**Authors:** Heidi Storm Vikke, Svend Vittinghus, Martin Betzer, Matthias Giebner, Hans Jørn Kolmos, Karen Smith, Maaret Castrén, Veronica Lindström, Marja Mäkinen, Heini Harve, Christian Backer Mogensen

**Affiliations:** 1 Department of Clinical Research, University of Southern Denmark, Odense, Denmark; 2 Medical Office, Falck Denmark A/S, Kolding, Denmark; 3 A&E Department, Sygehus Soenderjylland, Aabenraa, Denmark; 4 Department of Clinical Microbiology, Odense University Hospital, Odense, Denmark; 5 Ambulance Victoria, Centre for Research and Evaluation, Melbourne, Australia; 6 Department of Epidemiology and Preventive Medicine and Department Community Emergency Health and Paramedic Practice, Monash University, Melbourne, Australia; 7 Helsinki University Hospital, Department of emergency medicine and services, Helsinki University, Helsinki, Finland; 8 Karolinska Institutet, Department of Neurobiology, Care Sciences and Society, Division of Nursing & Academic EMS, Stockholm, Sweden; 9 Focused Research Unit in Emergency Medicine, Institute for Regional Health Research, University of Southern Denmark, Aabenraa, Denmark

## Abstract

**Background:**

Hand hygiene (HH), a cornerstone in infection prevention and control, lacks quality in emergency medical services (EMS). HH improvement includes both individual and institutional aspects, but little is known about EMS providers’ HH perception and motivations related to HH quality. Therefore, we aimed to investigate the HH perception and assess potential factors related to self-reported HH compliance among the EMS cohort.

**Methods:**

A cross-sectional, self-administered questionnaire consisting of 24 items (developed from the WHOs Perception Survey for Health-Care Workers) provided information on demographics, HH perceptions and self-reported HH compliance among EMS providers from Denmark.

**Results:**

Overall, 457 questionnaires were answered (response rate 52%). Most respondents were advanced-care providers, males, had > 5 years of experience, and had received HH training < 3 years ago. HH was perceived a daily routine, and the majority rated their HH compliance rate ≥ 80%. Both infection severity and the preventive effect of HH were acknowledged. HH quality was perceived important to colleagues and patients, but not as much to managers. Access to supplies, simple instructions and having or being “a good example” were perceived most effective to improve HH compliance. Self-reported HH compliance was associated with years of experience and perceptions of HCAI’s impact on patient outcome, HH’s preventive effect, organizational priority, HH’s importance to colleagues and patients, and the effort HH requires (*p* ≤ 0.05).

**Conclusion:**

Danish EMS providers acknowledged the impact of infections and the preventive effect of HH, and perceived access to HH supplies at the point of care, having or being “a good example” and simple instructions effective to improve HH compliance. Moreover, several behavioral-, normative- and control beliefs were associated with self-reported HH compliance, and thus future improvement strategies should be multimodal.

**Electronic supplementary material:**

The online version of this article (10.1186/s13049-019-0587-5) contains supplementary material, which is available to authorized users.

## Background

Healthcare-associated infections (HCAIs) occurring during care and treatment prolong hospitalization and increase the risk of readmission, consequently causing strain on both patients and society [1]. Proper hand hygiene (HH) is essential to prevent such infections, and information about why, how and when hand hygiene should be performed is widely available both nationally and internationally from for example; Statens Serum Institut (SSI) [2], Centers for Disease Control and Prevention (CDC) [3], the World Health Organization (WHO) [4], and the European Centre for Disease Prevention and Control (ECDC) [5].

According to WHO guidelines, HH implies hand wash with water and soap and/or hand rub. HH must be conducted in relation to the following five situations; before patient contact, before aseptic or clean procedures, after the risk of contact with body fluids, e.g., blood, secretions, after patient contact and after being in contact with patient-near surroundings. Also, HH must be performed before putting on gloves and after glove removal. Moreover, the use of gloves should be restrained to patient-care activities that involve risk of exposure to blood and all other body fluids, e.g., if in contact with mucous membranes and non-intact skin, and during contact precautions or in outbreak situations [4].

Despite HH being the most efficient way to prevent HCAI [4], it appears far from optimal in the emergency medical services (EMS). Studies have reported that EMS providers’ knowledge on the transmission of pathogens was insufficient [6] and that not all had received HH training [7]. Sub-optimal HH compliance [8, 9] and inappropriate use of gloves have also been reported [10, 11]. Lack of access to hygiene supplies at the point of care, time pressure and forgetfulness have been highlighted as critical barriers to appropriate HH in the EMS [12, 13], although research is limited. HH compliance is complicated and difficult to improve [14]. The WHO recommends a multimodal approach when improving compliance with hygiene practices in health-care settings [4]. In the nineties, behavioral theories and their applicability were assessed when searching for an appropriate framework to understand and target interventions to improve HH practices, while considering both individual and institutional factors. Focusing solely on individual components would be insufficient, and thus it has been recommended that interventions include both institutional climate, environmental limitations and individual elements [3]. The Theory of Planned Behavior is regularly applied, combining attitudes, subjective norms and perceived behavioral control to predict behavioral intentions, and thus a given HH behavior. Such an approach implies that the intention to perform HH is influenced by three central elements: beliefs about outcome causes (e.g., the perception of HCAI severity, or HHs preventive effect); beliefs about other people’s expectations (e.g., the perception of managers’, colleagues’ or patients’ expectations regarding HH performance) and control beliefs (e.g., the perceived effort HH demands). All elements are affected by demographics and individual life experience. Given substantial control over the behavior, e.g. HH, people are expected to carry out their intentions when the opportunity arises [14, 15]. Under such assumption, the intention to perform HH has been assessed by self-reported HH compliance and translated into presumed action [15]. This study aimed to investigate HH perception and factors related to self-reported HH compliance among the EMS cohort, using a behavioral theoretic approach.

## Methods

### Design and setting

An electronic survey including EMS providers from the frontline service in Denmark was conducted from November 2017 to February 2018. The providers were employed by a private organization covering approximately 70.000 patient courses, in rural and city areas, annually (precise data unavailable).

### Study population and survey content

A pre-determined study population of basic- and advanced-care providers received a questionnaire via e-mail during November 2017. A follow-up e-mail was forwarded to all participants (regardless of already responding) at least once during the three-month survey period to increase the response rate. Participation was anonymous to prevent distortion of answers towards more presumable acceptable opinions and behaviors [16]. Data was generated using an adjusted version of the WHO validated Health-Care Workers Hygiene Perception Survey [17]. Essential adjustments were the removal of questions not applicable to the EMS, and translation into Danish. The final version consisted of 24 questions regarding demographics, various measures’ effectiveness to improve HH compliance, and behavioral-, normative- and control beliefs. A pilot test was conducted, including 50 EMS providers, before data collection. The evaluation focused on, nonresponses (we had none), differences in response distribution (no outliers), and general usability of the questionnaire (all reports came back without missing answers within three days). A specialist group comprising the study researchers, EMS managers, and EMS providers discussed the outcome of the pilot including the essentials of every included question and concluded that no further adjustments were needed (see final questionnaire in Additional file 1).

### Statistics

Data was retrieved from a survey platform to an Excel data file, and then transferred to STATA 14 for analysis. Descriptive analyses were conducted calculating frequencies and proportions for nominal variables (dichotomous and ordinal), and median, 25th-, 75th percentiles and interquartile range for numeric variables (discrete). Correlation between numeric variables was assessed using Spearman’s test due to a negatively skewed distribution. Before an assessment of self-reported HH compliance and potential predictors, most variables were altered. The distribution of the variable “self-reported HH compliance” was negatively skewed, thus we created a dichotomous variable, defined by either good HH compliance (if the self-reported performance was ≥80%) or poor HH compliance (if the self-reported performance was < 80%), as described in a similar study involving doctors and nurses [15]. In addition, most 4-point Likert-scale variables had zero or few replies in some categories (“very low” or “low”), thus they were collapsed into one category, and several of the 7-point Likert scale variables had zero or few answers in both “negative” and “positive” categories, thus we chose to collapse the answers 1, 2 and 3 into one “negative” reply, left 4 as neutral, and collapsed 5, 6 and 7 into one “positive” reply.

Using the new dichotomic and ordinal variables, we analyzed potential relationships between self-reported HH compliance, demographics and various HH perceptions, using the Chi-squared test if the expected frequencies were 5 or above. If the expected frequencies were below 5, Fisher’s exact test was used. Odds Ratios (OR) were also calculated. In all tests, a *p*-value < 0.05 was considered statistically significant.

## Results

Of 876 distributed surveys, 457 were answered (response rate 52%). In total, 73% of the respondents were advanced-care providers, 90% were male, and 77% had more than five years of EMS experience. The qualification level and gender distribution of the respondents did not differ significantly from the study population (*p* > 0.05). Information about years of experience was not available for analysis. Table 1, presents the demographic data on the respondents and the study population, respectively.

**Table 1:** Demographic data on respondents and study population

	Respondents *n* = 457	Population *n* = 876	
Subject	Frequency (%)		*P*-value
Q2. Qualification level			
Basic-care	122 (27)	263 (30)	0.2033
Advanced-care	335 (73)	613 (70)	
Q3. Gender			
Male sex	413 (90)	782 (82)	0.5305
Q4. Years of experience			
< 1	11 (2)	*	
1–5	93 (20)	*	
6–10	77 (17)	*	
> 10	276 (60)	*	

Note: *Q* Question (for complete questions see questionnaire in Additional file 1), *Information unavailable

**Fig. 1:**
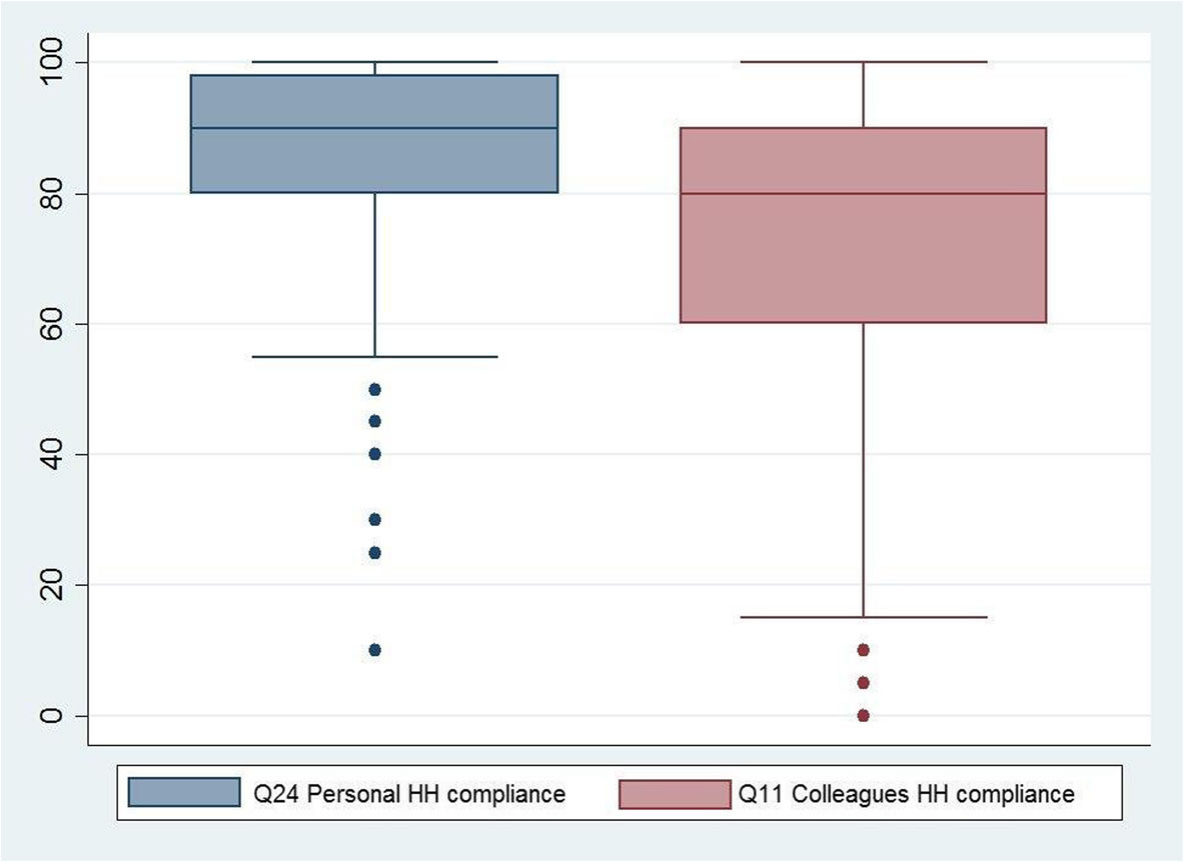
EMS providers’ self-reported HH compliance rate, and their perception of their colleagues’ HH compliance rate, in %

**Fig. 2:**
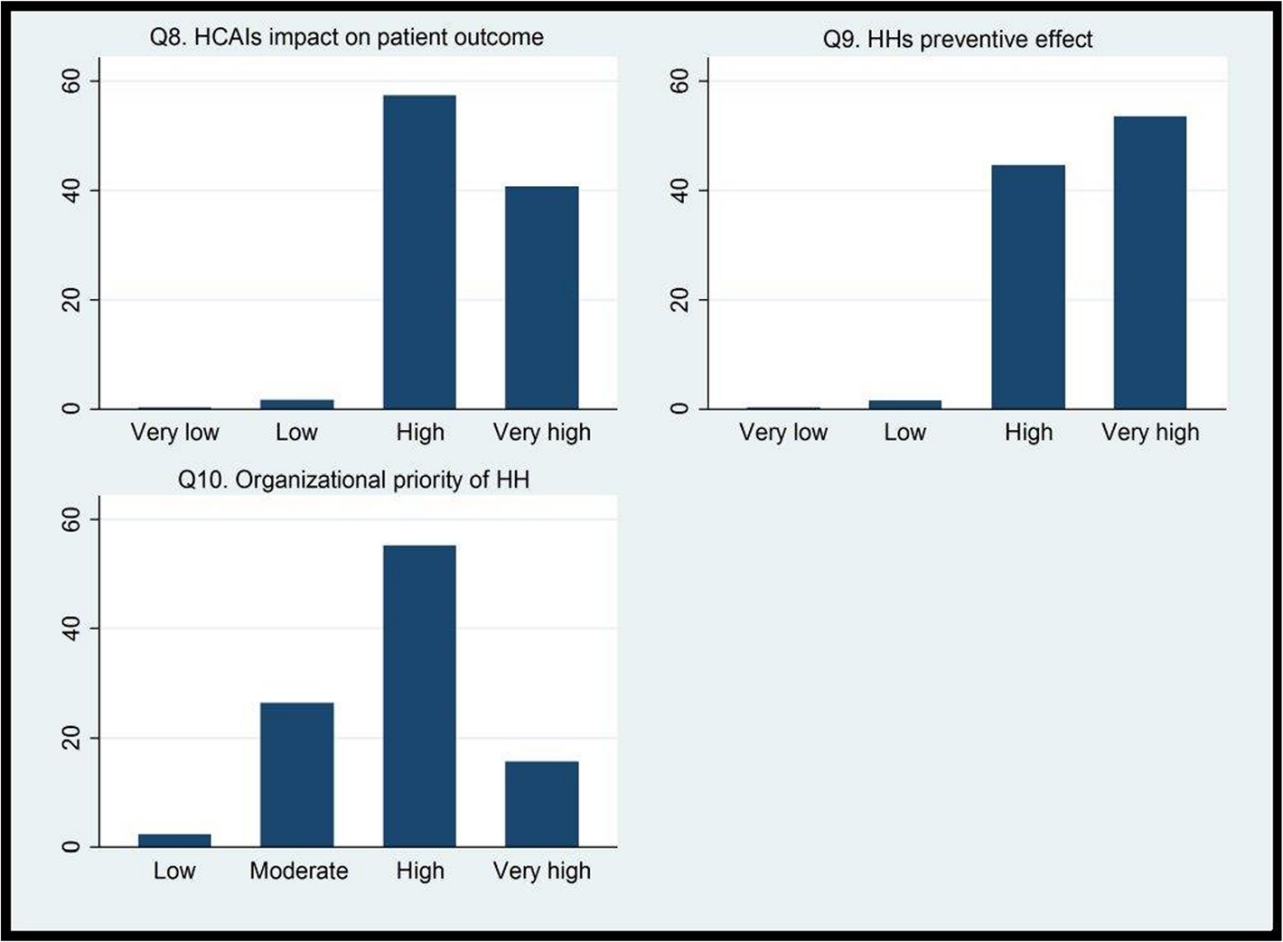
EMS providers’ perception of HCAI’s impact on patient outcome, the preventive effect of HH and the organizational priority of HH, in %

**Fig. 3:**
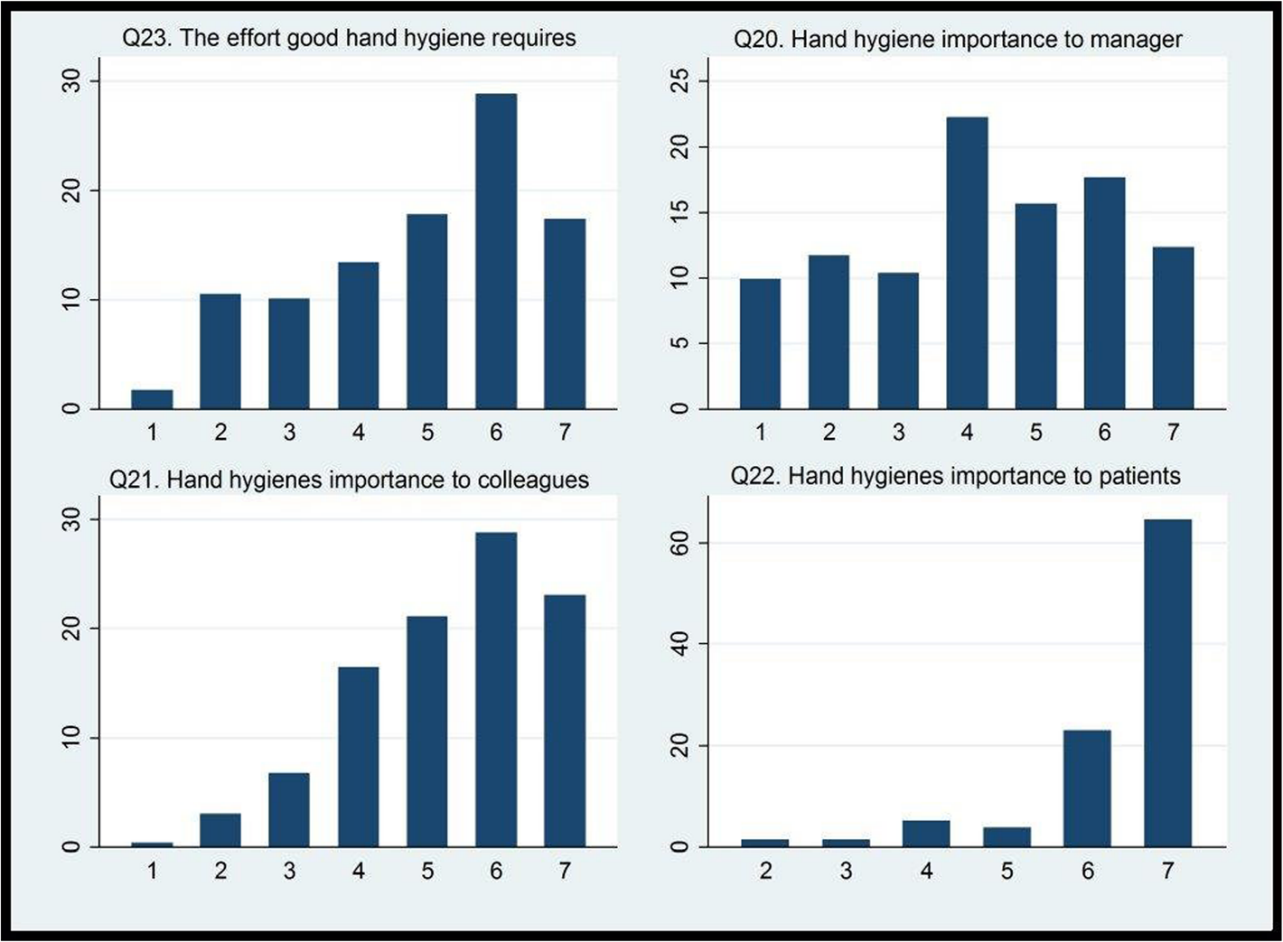
EMS providers’ perception of HH’s required effort, and its importance to managers, colleagues and patients (1: No effort, 7: A big effort, and 1: Not important, 7: Very important), in %

In total, 75% reported that they had received formal HH training less than three years ago, and 99% that they used alcohol-based hand rub or similar for HH, routinely. The perceived median HCAI rate was 25% (interquartile range 15–40%). The median self-reported HH compliance rate was 90% (interquartile range 80–98%), whereas the perceived median HH compliance rate among colleagues was 80% (interquartile range 60–90%) (Fig. 1. EMS providers’ self-reported HH compliance rate, and their perception of their colleagues’ HH compliance rate, in %). Most of the providers perceived the impact of HCAI on patient outcome (98%), the preventive effect of HH (98%) and the organizational priority of HH (71%) to be high or very high (Fig. 2. EMS providers’ perception of HCAI’s impact on patient outcome, the preventive effect of HH and the organizational priority of HH, in %). More than half of the providers believed that good HH required an extra effort (64%). Fewer perceived that the quality of their HH was important to their managers (47%), whereas, almost all believed that HH quality was important to their colleagues (73%) and to the patients (92%) (Fig. 3. EMS providers’ perception of HH’s required effort, and its importance to managers, colleagues and patients (1: No effort, 7: A big effort, and 1: Not important, 7: Very important), in %).

### EMS providers’ perception on measures effective to improve their HH compliance

**Fig. 4:**
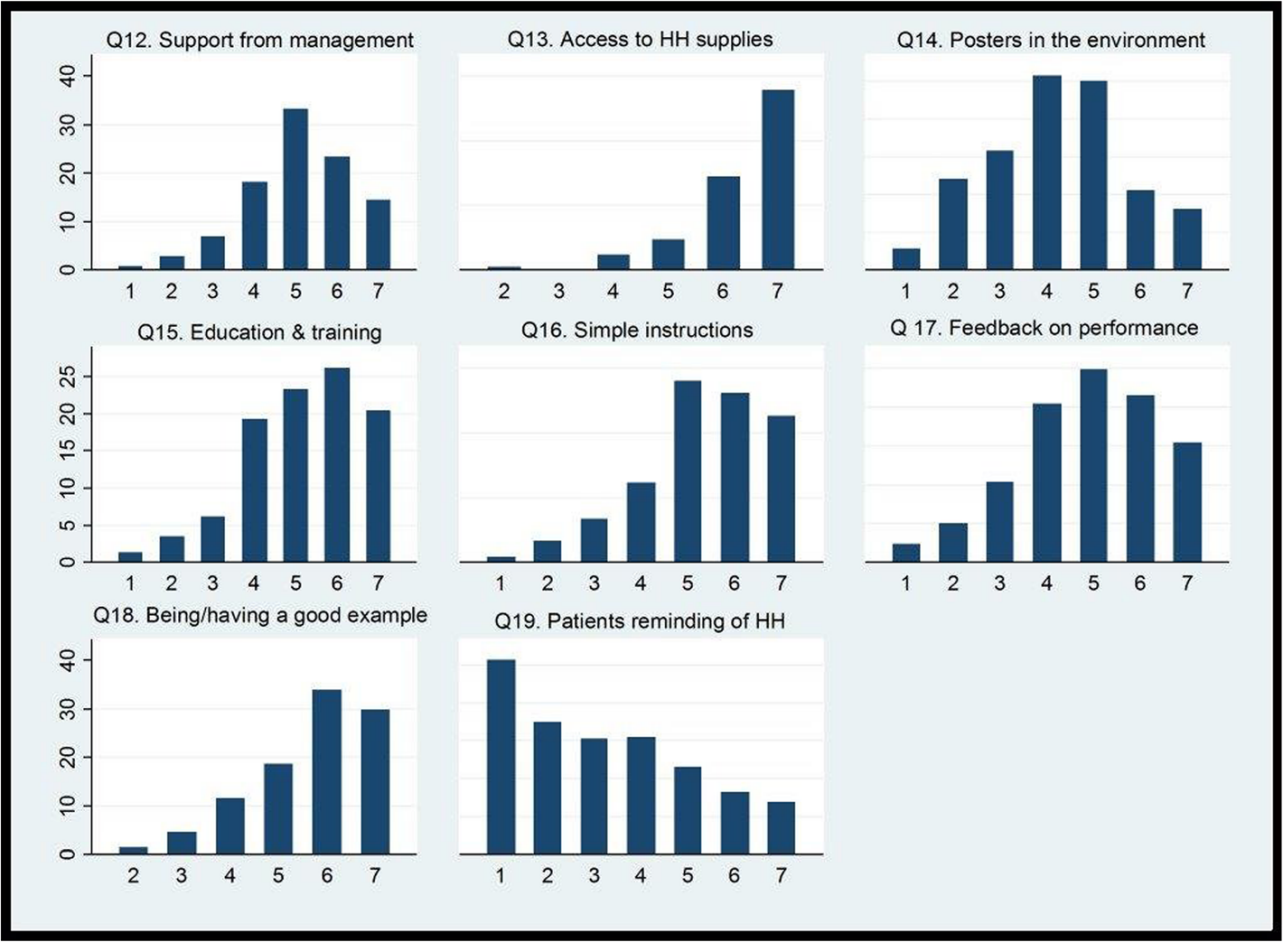
EMS providers’ perception of various measures’ effectiveness to improve HH compliance (1: Not effective, 7: Very effective), in %

Most of the providers believed that support from their managers (71%), access to supplies at point of care (94%), education and training (70%), simple and clear instructions (77%), feedback on performance (62%) and having or being “a good example” (82%) would be effective to improve their HH compliance. In contrast, reminder signboards (posters) in the environment (44%) and patient involvement (26%) were considered efficient by fewer providers (Fig. 4. EMS providers’ perception of various measures’ effectiveness to improve HH compliance (1: Not effective, 7: Very effective), in %).

### Factors related to perceived hand hygiene compliance of good quality

As reported, most of the providers perceived their personal HH to be of good quality (HH compliance rate > 80%), and they reported their compliance higher than their colleagues’ compliance. Moreover, the estimated personal compliance and estimated colleagues’ compliance were significantly correlated (Spearman’s rho 0.51, *p* = 0.000). The following factors were related to a self-reported HH of good quality: years of experience and perceptions of HCAI’s impact on patient outcome; HH’s preventive effect; organizational priority of HH; HH’s importance to colleagues and patients, and the effort required to perform good quality HH. Qualification level, gender, and perception of HH’s importance to managers were not related to self-reported HH compliance (Table 2).

**Table 2:** Factors related to perceived hand hygiene compliance of good quality among the EMS cohort

	Self-reported hand hygiene compliance rate			
	< 80%	≥ 80%	OR	*P*-value
Demographics/perceptions	Frequency (%)			
Q2. Qualification level				
Basic-care	17 (14)	104 (86)	1	0.542*
Advanced-care	54 (16)	275 (84)	0.8	
Q3. Gender				
Male	67 (17)	339 (83)	1	0.200*
Female	4 (9)	40 (91)	2	
Q4. Years of experience				
1–5 years	15 (15)	88 (85)	1	0.017*
6–10 years	20 (27)	55 (73)	0.5	
> 10 ten years	36 (13)	236 (87)	1.1	
Q8 HCAI’s impact on patient outcome is..				
Very low/low	2 (22)	7 (78)	1	0.035**
High	49 (19)	207 (81)	1.2	
Very high	20 (11)	165 (89)	2.4	
Q9. HH’s preventive effect is..				
Very low/low	3 (38)	5 (62)	1	0.026**
High	38 (19)	161 (81)	2.5	
Very high	13 (12)	213 (88)	4.6	
Q10. The organizational priority is..				
Very low/low	37 (28)	93 (72)	1	0.000*
High	30 (12)	219 (88)	2.9	
Very high	4 (6)	67 (94)	6.7	
Q20. HH’s importance to managers				
Not important	26 (18)	115 (82)	1	0.349*
Neutral	18 (18)	83 (82)	1	
Important	27 (13)	178 (87)	1.5	
Q21. HH’s importance to colleagues				
Not important	16 (35)	30 (65)	1	0.000*
Neutral	19 (26)	55 (74)	1.5	
Important	36 (11)	292 (89)	4.3	
Q22. HH’s importance to patients				
Not important	5 (36)	9 (64)	1	0.005**
Neutral	8 (33)	16 (67)	1	
Important	57 (14)	353 (86)	3.4	
Q23. Good quality HH requires..				
No extra effort	19 (19)	82 (81)	1	0.000*
Neutral	19 (32)	41 (68)	0.5	
Moderate to big effort	33 (12)	254 (88)	1.8	

Note. *Q* Question (for complete questions see questionnaire in appendix 1). *Chi-squared test, **Fisher’s exact test

## Discussion

This study is novel in assessing EMS providers’ HH perception and factors related to self-reported HH compliance.

One-third of the providers reported that they had not received any formal HH training within the last three years. The extent and severity of HCAIs and the preventive effect of HH were acknowledged. HH of good quality was perceived to require an extra effort and considered highly prioritized within the EMS organization by most. Few believed that the quality of their HH was important to their manager, whereas most believed that it was important to colleagues and patients. Access to supplies, having or being “a good example,” simple instructions, education and training, support from managers, and feedback on performance, were perceived effective to improve HH by most, whereas reminder signboards (posters) in the environment and patient involvement, were perceived useful by fewer. Finally, self-reported HH compliance was related to perceptions of HCAIs’ impact on patient outcome, HH’s preventive effect, organizational priority, HH’s importance to colleagues and to patients, and the effort required to perform good a HH.

Being the first study to investigate HH perception among EMS providers from a behavioral theoretic perspective, using the WHO scheme, makes it difficult to compare the present results with prior findings.

However, a substantial number of our respondents reported not to have received HH training within the last three years, and a similar lack of HH training has been reported in prior EMS studies [11–13, 18].

Sufficient training should be considered a management responsibility; therefore, it is a concern that most of the providers in our study did not perceive the quality of their HH essential to their managers.

Similar tendencies to lack of management prioritization have been reported elsewhere in the EMS, in the form of the absence of HH guidelines [7, 19], and lack of designated infection control officers [19] and quality assurance programs [20].

The fact that most of the providers, in our study, believed that having access to supplies at the point of care would improve their HH, is supported by results from a study reporting that EMS providers felt that their HH was compromised due to a lack of access to supplies at the point of care [11].

We found a relationship between self-reported HH compliance and peer-pressure (perceived organizational priority and perceived HH importance to colleagues and patients) similar to what has been reported among doctors and nurses in hospital settings [15]. Furthermore, we detected a relationship between self-reported HH compliance and HH’s importance to colleagues and a correlation between self-reported HH compliance and colleagues’ perceived HH compliance, further strengthening our presumption of peer pressure’s influence.

We also found EMS providers’ self-reported HH compliance related to their perception of the efforts required to perform good HH, a parameter that may be translated into self-efficacy [15]. This means that EMS providers’ self-efficacy may also be a factor to take into consideration when planning future interventions to improve HH.

To sum up, this study indicates that HH compliance among EMS providers is associated with multiple factors, e.g., organizational priority, peer-pressure, and available subjective and objective resources, which is in line with prior studies regarding HH behavior among comparable healthcare professions [21]. Therefore, we suggest a multimodal approach, in line with what is recommended by the WHO [4], involving cultural awareness, management support, prioritization of access to HH supplies at the point of care, and continuing HH training, when working on improving HH compliance among EMS providers. Additionally, we advocate that more research is conducted to elucidate the complexity of HH compliance among the EMS cohort.

## Limitations

A response rate of 52% makes responder bias a possibility and, although we found no significant difference between our respondents and the study population regarding qualification-level and gender distribution, the analysis included both respondents and non-respondents, due to anonymity, and thus we acknowledge the potential risk of selection bias (e.g., if the respondents were more interested in HH than the non-respondents). This must be taken into consideration when interpreting and generalizing our results.

Applying the pre-constructed WHO survey did not allow for an assessment of HH perceptions related to prior reported concerns of EMS providers’ focus on self-protection [8, 9]. Thus, we recommend that future studies seek to clarify this important area.

The evidence on behavior change theory’s potential to improve HH has not been extensively assessed, but several studies have applied the Theory of Planned Behavior in relation to HH in healthcare settings [15, 22, 23], and a recent review supports the potential [24].

Self-administration of the questionnaire might have distorted the findings towards more socially acceptable replies, especially in the light of peer-pressure’s influence on self-reported HH compliance. However, the fact that our survey was anonymous should have limited these biases.

It may also be considered a limitation that we measured the self-reported compliance instead of the actual HH compliance. However, self-reported compliance is an accepted surrogate due to the cost of large-scale observational studies. The probability of an overestimated self-reported behavior is known, and thus we must emphasize that the reported compliance should not be interpreted as a mimic of reality, but merely as a mean to assess factors related to HH compliance.

Finally, we were unable to perform adjusted analyses due to the small sample and negatively skewed distribution.

In the light of the present study’s limitations, we advocate that future studies take the risk of a skewed self-reported HH compliance, and the challenges related to enrollment of the responders into account during design and planning. Time and economy must support enrollment of a sample large enough to enable more complex analyses, to further strengthen the evidence on factors related to HH compliance. Also, in-depth interviews and group discussions may provide a more nuanced information, and thus support future understanding of the underlying needs regarding HH compliance within the EMS cohort.

## Conclusion

Many providers perceived HH a part of their regular routines, and they acknowledged both the extent and the severity of HCAI, along with the preventive effect of HH. Access to HH supplies at the point of care, training, and simple instructions were top priorities among measures to improve HH compliance. Also, organizational priority, peer-pressure, and self-efficacy were related to self-reported HH compliance. Thus, we recommend a multimodal approach involving cultural awareness, management support, and promotion, evaluation and support of access to HH supplies at the point of care, along with continuing HH training and quality monitoring in future efforts to improve hand hygiene among the EMS cohort.

## Additional file

Additional file 1: Final questionnaire. (PDF 401 kb)

## Abbreviations

CDC: Centers for Disease Control and Prevention
ECDC: European Centre For Disease Prevention and Control
EMS: Emergency medical service
HCAI: Healthcare-associated infection
HH: Hand hygiene
SSI: Statens Serum Institut
WHO: World Health Organization
