# Supplementary material for: “Hand hygiene perception and self-reported hand hygiene compliance among emergency medical service providers: a Danish survey”
Source: Scand J Trauma Resusc Emerg Med. 2019 Feb 5;27:10. doi: 10.1186/s13049-019-0587-5 (PMC6362569; doi:10.1186/s13049-019-0587-5)
Supplement: Supplementary file 1 — Final questionnaire. (PDF 401 kb) [file 13049_2019_587_MOESM1_ESM.pdf]

Questionnaire on perception of hygiene the emergency medical services (EMS)

This study is the third and final sub study in a Danish PhD about prehospital hygiene. The initial two projects investigated bacterial contamination in Danish ambulances and hygiene compliance among EMS staff in Finland, Sweden, Australia and Denmark. The results show a great improvement potential, and to secure that future interventions take EMS staffs perception in to account, we need your answers to a number of questions regarding hand hygiene.

On behalf of the study group
Kind regards
Heidi Vikke

Question	Answers
1. Affiliation	1. Finland<br>2. Sweden<br>3. Australia
2. Provider care-level	1. ETA<br>2. EMT<br>3. Paramedic<br>4. Registered nurse
3. Gender	1. Male<br>2. Female
4. Years of EMS experience	1. < 1<br>2. 1-5<br>3. 6-10<br>4. > 10
5. Have you received formal training in hand hygiene in the last three years?	1. Yes<br>2. No
6. Do you routinely use alcohol-based hand rub or similar for hand hygiene?	1. Yes<br>2. No
7. What is the average percentage of hospitalized patients who will develop an infection?	1. From 0-100%<br>2. I don't know
8. What is the impact of a health care-associated infection on a patient's clinical outcome?	1. Very Low<br>2. Low<br>3. High<br>4. Very high
9. What is the effectiveness of hand hygiene in preventing health care-associated infection?	1. Very Low<br>2. Low<br>3. High<br>4. Very high
10. Among all patient safety issues, how important is hand hygiene in your organization?	1. Low priority<br>2.Moderate priority<br>3. High priority<br>4. Very high priority
11. On average, in what percentage of situations requiring hand hygiene do your colleges actually perform hand hygiene, either by hand rubbing or hand washing?	1. From 0-100%<br>2. I don't know

12. How effective do you think, leaders and senior managers at your institution supporting hand hygiene, would be to improve hand hygiene permanently in your institution?	Each question answered by points from 1-7 (from Not effective to Very effective)
13. How effective do you think, alcohol-based hand rub always available at each point of care, would be to improve hand hygiene permanently in your institution?	Each question answered by points from 1-7 (from Not effective to Very effective)
14. How effective do you think, hand hygiene posters displayed at point of care as reminders, would be to improve hand hygiene permanently in your institution?	Each question answered by points from 1-7 (from Not effective to Very effective)
15. How effective do you think, regular education and training, would be to improve hand hygiene permanently in your institution?	Each question answered by points from 1-7 (from Not effective to Very effective)
16. How effective do you think, simple and clear instructions for hand hygiene, would be to improve hand hygiene permanently in your institution?	Each question answered by points from 1-7 (from Not effective to Very effective)
17. How effective do you think, regular feedback on hand hygiene performance, would be to improve hand hygiene permanently in your institution?	Each question answered by points from 1-7 (from Not effective to Very effective)
18. How effective do you think, being a good example for your colleagues, would be to improve hand hygiene permanently in your institution?	Each question answered by points from 1-7 (from Not effective to Very effective)
19. How effective do you think patients reminding EMS workers to perform hand hygiene would be to improve hand hygiene permanently in your institution?	Each question answered by points from 1-7 (from Not effective to Very effective)
20. What importance does the head of your department attach to the fact that you perform optimal hand hygiene?	Question is answered by points from 1-7 (from no importance to very high importance)
21. What importance do your colleagues attach to the fact that you perform optimal hand hygiene?	Question is answered by points from 1-7 (from no importance to very high importance)
22. What importance do patients attach to the fact that you perform optimal hand hygiene?	Question is answered by points from 1-7 (from no importance to very high importance)
23. How do you consider the effort required by you to perform good hand hygiene when caring for patients?	Question is answered by points from 1-7 (from no effort to A big effort)
24. On average, in what percentage of situations requiring hand hygiene do you actually perform hand hygiene, either by hand rubbing or hand washing?	From 0-100%

Spørgeskema om hygiejneforståelse i Dansk ambulancetjeneste

Denne undersøgelse er det tredje og sidste delprojekt i en dansk Ph.d. om præhospital hygiejne. De første to projekter har undersøgt hhv. bakteriel forurening i dansk ambulancetjeneste og håndhygiejne blandt ambulancepersonale i Finland, Sverige, Australien og Danmark. Resultaterne viser at der er et forbedringspotentiale og i den henseende bør fremtidige initiativer være baseret på kendskab til international ambulancepersonales hygiejneforståelse, og derfor har vi brug for jeres svar på en række spørgsmål om håndhygiejne.

På forhånd tak og med venlige hilsner
Heidi Vikke & Matthias Giebner
Svend Vittinghus & Martin Betzer

Spørgsmål	Svarmuligheder
1. Ansættelsessted	1. Falck<br>2. Responce
2. Behandlerniveau	1. Assistent<br>2. Behandler<br>3. Paramediciner
3. Køn	1. Mand<br>2. Kvinde
4. Erfaringsgrundlag (antal år)	1. < 1<br>2. 1-5<br>3. 6-10<br>4. > 10
5. Har du modtaget undervisning i håndhygiejne inden for de seneste tre år?	1. Ja<br>2. Nej
6. Anvender du håndsprit rutinemæssigt?	1. Ja<br>2. Nej
7. Hvor mange patienter tror du, der udvikler en infektion under deres indlæggelse (i %)?	1. Fra 0-100%<br>2. Ved ikke
8. Hvor stor en betydning tror du, at infektion har for patientens sygdomsforløb?	1. Meget lille<br>2. Lille<br>3. Stor<br>4. Meget stor
9. Hvor stor er effekten v. håndhygiejne i forbindelse med forebyggelse af infektion?	1. Meget lille<br>2. Lille<br>3. Stor<br>4. Meget stor
10. Blandt alle patientsikkerhedstiltag, hvordan prioriteres håndhygiejne i din organisation?	1.Lav prioritet<br>2. Moderat prioritet<br>3.Høj prioritet<br>4. Meget høj prioritet
11. I hvor stor en andel af situationer, der kræver håndhygiejne vurderer du, at dine kolleger enten vasker hænder eller bruger håndsprit (i %)?	1. Fra 0-100%<br>2. Ved ikke

12. Hvor effektiv vurderer du, at ledelsesmæssig støtte og promovering af håndhygiejne er til at forbedre håndhygiejnen i din organisation?	Spørgsmålet besvares ved point fra 1-7 (fra ikke effektivt til meget effektivt)
13. Hvor effektiv vurderer du, at hånd sprit altid er tilgængeligt under patienthåndtering er til at forbedre håndhygiejnen i din organisation?	Spørgsmålet besvares ved point fra 1-7 (fra ikke effektivt til meget effektivt)
14. Hvor effektiv vurderer du, at plakater der synliggør og påminder om håndhygiejne er til at forbedre håndhygiejnen i din organisation?	Spørgsmålet besvares ved point fra 1-7 (fra ikke effektivt til meget effektivt)
15. Hvor effektiv vurderer du, at regelmæssig undervisning i håndhygiejne er til at forbedre håndhygiejnen i din organisation?	Spørgsmålet besvares ved point fra 1-7 (fra ikke effektivt til meget effektivt)
16. Hvor effektiv vurderer du, at simple og klare håndhygiejneinstrukser let tilgængelige for alle medarbejdere, er til at forbedre håndhygiejnen i din organisation?	Spørgsmålet besvares ved point fra 1-7 (fra ikke effektivt til meget effektivt)
17. Hvor effektiv vurderer du, at regelmæssigt feedback på håndhygiejne præstation er til at forbedre håndhygiejnen i din organisation?	Spørgsmålet besvares ved point fra 1-7 (fra ikke effektivt til meget effektivt)
18. Hvor effektiv vurderer du, at du altid selv udfører håndhygiejne jf. instrukser (at du er et godt eksempel for dine kolleger) er til at forbedre håndhygiejnen i din organisation?	Spørgsmålet besvares ved point fra 1-7 (fra ikke effektivt til meget effektivt)
19. Hvor effektiv vurderer du, at patienter der påminder ambulancepersonalet om håndhygiejne er til at forbedre håndhygiejnen i din organisation?	Spørgsmålet besvares ved point fra 1-7 (fra ikke effektivt til meget effektivt)
20. Hvor stor betydning har det for din nærmeste leder, at du udfører korrekt håndhygiejne?	Spørgsmålet besvares ved point fra 1-7 (fra ingen betydning til meget stor betydning)
21. Hvor stor betydning har det for dine kolleger, at du udfører korrekt håndhygiejne?	Spørgsmålet besvares ved point fra 1-7 (fra ingen betydning til meget stor betydning)
22. Hvor stor betydning har det for patienterne, at du udfører korrekt håndhygiejne?	Spørgsmålet besvares ved point fra 1-7 (fra ingen betydning til meget stor betydning)
23. Hvordan vurderer du den indsats, der kræves af dig i forhold til at udføre korrekt hånd hygiejne under patient håndtering?	Spørgsmålet besvares ved point fra 1-7 (fra ingen indsats til en stor indsats)
24. I hvor stor en andel af situationer, der kræver håndhygiejne udfører du enten håndvask eller bruger håndsprit (i %)?	0-100%
